# Stiff left atrial syndrome as another cause for heart failure with preserved ejection fraction and normal BNP levels

**DOI:** 10.1007/s12471-016-0924-5

**Published:** 2016-12-15

**Authors:** C. M. H. B. Lucas

**Affiliations:** Alrijne Zorggroep, Leiderdorp, The Netherlands

In a recent issue of the Netherlands Heart Journal, Meijers et al. describe in their paper a subgroup of patients of the COACH study presenting with heart failure with preserved ejection fraction and low levels of natriuretic peptides [1]. Major differences between patients with elevated and normal BNP (NT-proBNP) levels were found in BMI which has been shown before to influence the levels of this biomarker. However, there might be another reason for this low level of BNP; namely, the fact that the cause of the heart failure symptoms is not related to left ventricular function but is due to abnormalities in left atrial function and structure. This clinical picture was described for the first time in 1988 and has been called the ‘Stiff left atrial syndrome’ [2]. In this syndrome it has been shown that a primary increase in filling pressures in the left atrium results in secondary elevated pulmonary pressures. Left ventricular end-diastolic filling pressures appeared to be normal. This pattern therefore fits both the clinical picture of heart failure as well as the neurohormonal patterns as described in the paper by Meijers et al. That is, normal BNP/NT-proBNP and troponin I, which are biomarkers released in the circulation when abnormalities are present in the left ventricular wall, either by elevated filling pressures, wall stress or ischaemia. This is not the case in the stiff left atrial syndrome. All other mentioned biomarkers are released into the circulation as a result of other haemodynamic derangements such as diminished kidney perfusion. The syndrome of the stiff left atrial syndrome was first found and described in patients who had chronic atrial fibrillation and previous mitral valve repair. Fibrosis of the left atrium appeared to play a role in this. The syndrome itself might be more prevalent than we anticipate as a recent single-centre study demonstrated a significant number of patients presenting with this form of heart failure [3]. Recently renewed attention has been given to this syndrome as an unwanted result of surgery or radiofrequency ablation for atrial fibrillation [4]. One of the causes might be progressive fibrosis or shrinking of the left atrial wall due to the procedure. In my personal opinion this syndrome requires further research both investigating its prevalence as well as possible causes. The latter might have consequences for the way current techniques are employed to treat atrial fibrillation.
